# Insurance, legal, and financial hardships of childhood and adolescent cancer survivors—a systematic review

**DOI:** 10.1007/s11764-024-01710-3

**Published:** 2024-11-29

**Authors:** Martina Ospelt, Pauline Holmer, Eva Maria Tinner, Luzius Mader, Manya Hendriks, Gisela Michel, Sonja Kälin, Katharina Roser

**Affiliations:** 1 Faculty of Health Sciences and Medicine, University of Lucerne, Lucerne, Switzerland; 2 Division of Pediatric Hematooncology, Inselspital, University Hospital Bern, Bern, Switzerland; 3 University Center of Internal Medicine, Kantonsspital Baselland, Liestal, Switzerland; 4 Cancer Registry Bern Solothurn, University of Bern, Bern, Switzerland

**Keywords:** Socio-bureaucratic hardships, Financial hardships, Insurance hardships, Legal hardships, Childhood cancer, Systematic review

## Abstract

**Purpose:**

Childhood and adolescent cancer survivors (CACS) experience medical and psychosocial adverse effects. Attention widens to include issues such as socio-bureaucratic hardships. This systematic review synthesized the available evidence on insurance, legal, and financial hardships to better understand the broader picture of socio-bureaucratic hardships as distinct but interrelated types of hardships.

**Methods:**

A systematic search of PubMed, Scopus, CINAHL, and PsycINFO was conducted for publications related to childhood and adolescent cancer; survivors; and insurance, legal, and financial hardships. Narrative data synthesis was performed on the extracted data.

**Results:**

This review included *N* = 58 publications, originating from 14 different countries, most from the last decade (*n* = 39). We found that a considerable proportion of CACS experience insurance and financial hardships, including foregoing medical care due to financial constraints, problems paying medical bills, and difficulties accessing loans or insurances. Legal hardships, such as workplace discrimination, were less frequently investigated and reported.

**Conclusions:**

This systematic review highlights the many interrelated socio-bureaucratic hardships faced by CACS. It is important that these hardships are not underestimated or neglected. Our findings can serve as a basis for enhancing and expanding supportive care services and help inform collaborative efforts from research, policy, and practice.

**Implications for Cancer Survivors:**

This review emphasizes the importance of recognizing and addressing the socio-bureaucratic challenges that extend beyond medical care. Survivors should be informed about available options and be aware of their legal rights to identify instances of injustice and seek appropriate support.

**Supplementary Information:**

The online version contains supplementary material available at 10.1007/s11764-024-01710-3.

## Introduction

Navigating the health care system has been shown to be challenging [1]. For those who have been affected by childhood and adolescent cancer, it can be even more difficult [2]. Survivors of childhood and adolescent cancer (CACS) face difficulties with insurance, legal, and financial hardships. Insurance hardships as a consequence of cancer may include difficulties obtaining and maintaining insurance [3–5] or paying higher premiums [6, 7]. Discrimination and limited access to public services have been described as legal hardships [8]. In relation to cancer, this may involve more difficult access to appropriate education, employment, rehabilitation, dental plans, disability benefits, and insurance [8]. Financial hardships can be specified as experiencing financial distress due to the cancer diagnosis or treatment [9]. Financial burden can be categorized into material, behavioral, and psychological hardship [10], such as the inability to pay for medical care, delaying or forgoing care, and worries regarding finances and insurance coverage [10, 11].

CACS report higher out-of-pocket medical expenses [12, 13], are more often uninsured [4, 13], face difficulties obtaining life insurance [14, 15], and have a higher uptake of social security or disability benefits [16]. Insurance and financial hardships are shown to potentially exacerbate or cause physical and psychological harm to CACS including stress, anxiety, and impaired sleep [4, 13, 17]. Moreover, the physical and psychological late effects associated with childhood and adolescence cancer may further contribute to insurance, legal, or financial hardships. Additional risk factors, such as low income, pre-existing financial difficulties, unemployment, or lack of a social network, may also increase the risk of encountering these hardships [10, 17–19].

While medical and psychosocial adverse effects have been extensively investigated, less attention has been paid to socio-bureaucratic hardships experienced by CACS [20, 21]. This systematic review explored insurance, legal, and financial hardships as three distinct but interrelated types of hardships. With this systematic review, we aimed to provide a comprehensive overview of socio-bureaucratic hardships faced by CACS. More specifically, we aimed to describe (i) evidence on insurance, legal, and financial hardships reported by CACS and (ii) risk factors associated with the respective hardships. By covering three intertwined and mutually influencing topics, rather than examining them separately, we aimed to gain a better understanding of the broader picture of socio-bureaucratic hardships experienced by CACS and associated risk factors.

## Methods

This review complies with the Preferred Reporting Items for Systematic Reviews and Meta-Analysis (PRISMA) guidelines [22] and was preregistered on PROSPERO (No. CRD42023423759).

### Literature search

We systematically searched the electronic databases PubMed, CINAHL, Scopus, and PsychINFO using the four blocks *insurance*, *legal*, *or financial hardships*; *survivors or parents*; *childhood and adolescence*; and *cancer* (Supplemental appendix A). The search blocks were adapted for each database. The databases searched were selected based on their different thematic focus to identify relevant publications from relevant areas. Eligible for this review were peer-reviewed publications that examined the insurance, legal, or financial hardships of CACS. Although the systematic search included both survivors and parents, the current review only includes publications focused on survivors. Hardships experienced by parents are discussed in another publication. The search was conducted on March 16, 2023, and updated on June 24, 2024.

### Selection criteria

We included peer-reviewed, original research publications on insurance, legal, and financial hardships of adult CACS. At least 2 years had to have passed since diagnosis and survivors had to have received their cancer diagnosis before the age of 18 (≥ 75% of sample, or separate analyses). As insurance, legal, and financial hardships, we have considered difficulties or problems experienced by CACS in these areas that result from childhood and adolescent cancer and/or cancer-related consequences. The publication selection criteria (Supplemental appendix B) were applied hierarchically to select eligible publications.

### Publication screening

In a first step, duplicates were manually removed using the non-automated web tool Rayyan (https://rayyan.ai/). In a second step, titles and abstracts of all identified publications were independently screened by at least two reviewers (MO, PH, SK, KR). Rayyan was used to track reviewers’ decisions. Then full texts of potentially relevant publications were obtained and independently reviewed in a separate Rayyan bibliography by at least two reviewers (MO, PH, SK, KR). Disagreements were resolved by discussion among the reviewers (MO, PH, SK, KR).

### Data extraction

Data extraction was conducted by the first author (MO) and double checked by another researcher (PH, SK, KR). Study characteristics (e.g., country, language, study design, sample size), survivor characteristics (e.g., age at study, cancer diagnosis, time since diagnosis), and, if applicable, information on comparison groups were extracted into a predefined data extraction sheet. We extracted detailed information on insurance, legal, and financial hardships and risk factors.

### Quality assessment

To assess the methodological quality of the included publications, we used the Quality Assessment with Diverse Studies (QuADS) tool [23]. The tool is suitable for qualitative, quantitative, and mixed-methods study designs and has shown substantial reliability and validity [23]. The QuADS tool includes 13 evaluation criteria on a scale from 0 (no mention at all) to 3 (detailed information). Two reviewers each (MO, PH, SK, KR) independently rated all included publications, and a percentage of the maximum score was calculated for each publication.

### Data synthesis

To account for the inclusion of both quantitative and qualitative studies in this review, we chose to use narrative synthesis to analyze and report the findings [24].

## Results

### Publication selection

**Fig. 1:**
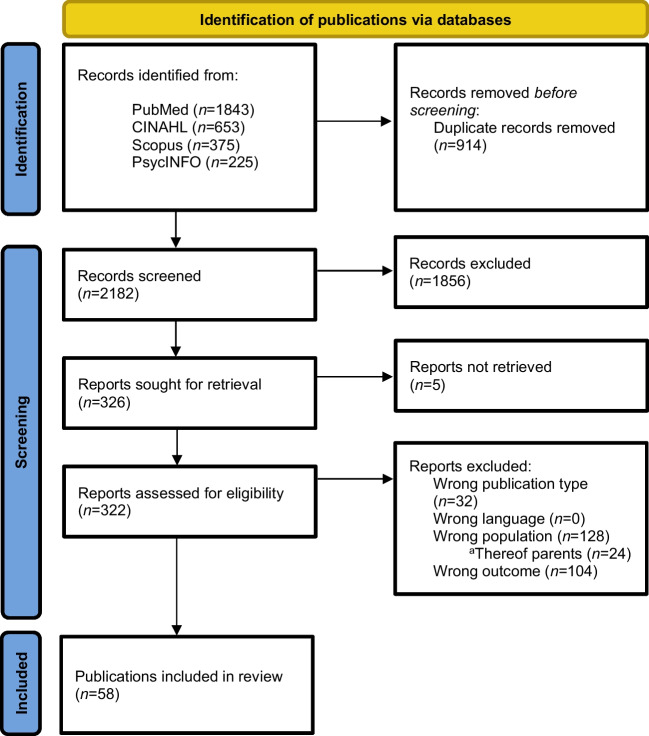
PRISMA flow diagram. ^a^Combined search for survivors and parents. The hardships experienced by parents are discussed in another publication

We identified 3096 records in the four searched databases: PubMed (*n* = 1843), CINAHL (*n* = 653), Scopus (*n* = 375), and PsycINFO (*n* = 225). After duplicate removal (*n* = 914), 2182 records were included in the title and abstract screening. Thereof, 322 full texts were obtained and screened. Finally, *N* = 58 publications were included in this review focusing on CACS. The PRISMA flow chart displays the detailed procedure for selecting eligible publications (Fig. 1).

### Publication characteristics

Most of the publications were quantitative studies (*n* = 48), fewer qualitative (*n* = 6), and mixed methods (*n* = 4). Publications originated in a total of 14 countries. All but three publications originated from countries with a high sociodemographic index (two high-middle, one middle-income) [25]. Most publications were from the US (*n* = 31) and Northern or Western European countries (*n* = 19), with fewer from Asia (*n* = 4), the middle east (*n* = 2), and Canada (*n* = 2). Publication years ranged from 2000 to 2024, with more than two-thirds published in the last decade (*n* = 39) and nearly 40% published since 2020 (*n* = 23). An overview of included publications is provided in Table 1.

**Table 1:** Overview of included publications

Authors, year ^[REF]^	Country	Study design	Number of survivors	Sex/gender distribution	Mean age at diagnosis in years (SD/range)	Mean time since diagnosis in years (SD/range)	Mean age at study in years (SD/range)	Comparison group	Hardship domains	Reported hardships
Al-Rawashdeh et al., 2024 [27]	Jordan	Quantitative and qualitative	*N* = 297	Female (41%) Male (59%)	≤ 5 years: 60 (20%) 6–11 years: 74 (25%) ≥ 12 years: 163 (55%)	≤ 5 years: 41 (14%) 6–10 years: 110 (37%) > 10 years: 146 (49%)	22.4 (3.5/NR)	No	Financial	• Financial needs • Socioeconomic challenges • Needs regarding money to cover living expenses, to treat other illnesses and to treat side effects of cancer and its treatments
Baecklund et al., 2022 [28]	Sweden	Quantitative	*N* = 1305	Female (48%) Male (52%)	0–4 years: 432 (33%) 5–9 years: 313 (24%) 10–14 years: 350 (27%) 15–17 years: 210 (16%)	NR	NR (NR/20–25)	Yes (matched controls)	Financial Insurance	• Higher proportions of disability pension • SADP more common
Baedke et al., 2021[29]	USA	Quantitative	*N* = 3964	Female (48%) Male (52%)	NR	5–9 years: 139 (3%) 10–19 years: 1237 (31%) 20–29 years: 1336 (34%) 30–39 years: 910 (23%) 40–49 years: 316 (8%) ≥ 50 years: 26 (1%)	< 20 years: 165 (4%) 20–29 years: 1427 (36%) 30–39 years: 1302 (33%) 40–49 years: 797 (20%) ≥ 50 years: 273 (7%)	Yes (ethnic subgroups within)	Financial Insurance	• Forgoing needed medical care (due to finances) • Losing insurance
Bejarano-Quisoboni et al., 2022[30]	France	Quantitative	*N* = 5319	Female (45%) Male (55%)	0–1 years: 937 (18%) 2–4 years: 1034 (19%) 5–9 years: 1088 (21%) 10–14 years: 1130 (21%) ≥ 15 years: 1130 (21%)	NR	< 20 years: 550 (10%) 20–30 years: 1979 (37%) 31–40 years: 1978 (36%) 41–50 years: 753 (14%) ≥ 51 years: 159 (3%)	No	Financial	• High health care expenditures
Bejarano-Quisoboni et al., 2024 [31]	France	Quantitative	*N* = 5353	Female (46%) Male (54%)	0–1 years: 1277 (24%) 2–4 years: 1253 (23%) 5–9 years: 1183 (22%) 10–14 years: 1084 (20%) ≥ 15 years: 556 (11%)	24.7 (NR/17–31)	< 20 years: 818 (15%) 20–30 years: 1814 (34%) 31–40 years: 1699 (32%) ≥ 41 years: 1022 (19%)	Yes (general population)	Financial	• High excess health care expenditures
Boman et al., 2010[32]	Sweden	Quantitative	*N* = 1716	Female (49%) Male (51%)	NR	NR	31.6 (NR/NR)	Yes (general population)	Financial	• Economic compensation due to disability • Lower net income
Buchbinder et al., 2023[33]	USA and Canada	Quantitative	*N* = 2844	Female (52%) Male (48%)	NR	NR (NR/17.7–48.7)	18–30 years: 444 (16%) 30–39 years: 1056 (37%) > 40 years: 1344 (47%)	Yes (siblings)	Financial	• Financial hardship • Material, behavioral, and psychological financial hardship
Carlson–Green, 2009[6]	USA	Qualitative	*N* = 11	NR	≥ 5 years of survivorship	NR	28.4 (NR/23–33)	No	Financial Insurance	• Insurance concerns: denials because of preexisting conditions, expense of having to pay more for their insurance premium or having enormous deductibles • Insurance expenses preventing CCS from, e.g., saving for down payments or putting away money for retirement
Chae et al., 2020[34]	South Korea	Quantitative	*N* = 7317	Female (46%) Male (54%)	0–4 years: 2220 (30%) 5–9 years: 1545 (21%) 10–14 years: 2048 (28%) 15–17 years: 1504 (21%)	NR	NR	No	Financial	• Medical costs
Chan et al., 2020[35]	China	Quantitative	*N* = 614	Female (59%) Male (41%)	NR	14.1 (6.8/NR)	21.9 (5.6/NR)	Yes (siblings)	Insurance	• Insurance coverage
Clemens et al., 2017[7]	The Netherlands	Quantitative	*N* = 658	Female (44%) Male (56%)	Median 6.2 (NR/0.01–17.8)	Median 15.6 (NR/3.2–43.7)	Median 23.5 (NR/14.6–52.3)	Yes (platinum with non-platinum treatment)	Insurance	• Problems obtaining insurance • Higher insurance premiums
Crom et al., 2007[36]	USA	Quantitative	*N* = 1437	Female (50%) Male (50%)	Median 6.7 (NR/0.1–21.1)	Median 21.1 (NR/10–39.2)	Median 29.7 (NR/18.2–55.3)	Yes (general population)	Insurance	• Denial of insurance • Difficulty/delay in obtaining medical care
Dumas et al., 2017[14]	France	Quantitative	*N* = 1920	Female (47%) Male (53%)	0–4 years: 1041 (54%) 5–9 years: 446 (23%) 10–14 years: 366 (19%) ≥ 15 years: 67 (4%)	31.2 (7.1/NR)	36.3 (8.0/NR)	No	Financial Insurance Legal	• Difficulties when trying to access loans • Difficulties in accessing insurance for a home loan (including rejection, higher premiums, and exclusions) • Difficulties in accessing a personal loan • Refusal to insure survivors because of their history of pediatric cancer
Fair et al., 2021[11]	USA and Canada	Quantitative	*N* = 698	Female (55%) Male (45%)	0–5 years: 404 (46%) 6–10 years: 104 (19%) 11–15 years: 109 (20%) 16–20 years: 81 (15%)	Median 28.8 (NR/23.1–41.7)	22–29 years: 214 (11%) 30–39 years: 228 (42%) ≥ 40 years: 256 (47%)	Yes (siblings)	Financial	• Medical financial hardship • Material financial hardship including conditions that arise from medical expenses • Behavioral financial hardship including coping behaviors to manage medical expenses • Psychological financial hardship resulting from worries about medical expenses and insurance
Fauer et al., 2024[37]	USA	Quantitative	*N* = 3475	Female (52%) Male (48%)	Median 8 (NR/4–13)	NR	Median 39.1 (NR/33.4–46.6)	Yes (siblings)	Financial	• Financial hardship
Fiala, 2021[38]	USA	Quantitative	633	Female (71%) Male (29%)	NR	NR (NR/0–61)	NR (NR/21–63)	Yes (pre vs. post ACA) matched with non-cancer controls (= peers without cancer)	Financial Insurance	• Difficulty with health care affordability • More medical non-adherence due to costs than their peers • Foregoing needed health care
Gunnes et al., 2016[39]	Norway	Quantitative	*N* = 2139	Female (56%) Male (44%)	NR	NR	NR	Yes (general population)	Financial	• Financial dependence • Elevated risk of receiving governmental financial assistance
Guy et al., 2016[40]	USA	Quantitative	*N* = 239	Female (57%) Male (43%)	NR	≥ 20 years: (72%)	18–34 years: 92 (42%) 35–50 years: 72 (33%) 51–64 years: 32 (12%) ≥ 65 years: 43 (13%)	Yes (general population)	Financial Insurance	• Productivity loss • Household productivity loss • Insurance coverage
Hendriks et al., 2021[3]	Switzerland	Qualitative	*N* = 28	Female (68%) Male (32%)	9.3 (NR/0.5–16)	Time since end of treatment: 19.1 (NR/2–38)	31.4 (NR/18–55)	No	Insurance Legal	• Concerns about many different facets of insurance • Difficulties with reimbursements • Challenges with DI process • Difficulties with supplementary health insurance or life insurance • Limited eligibility/flexibility for supplementary insurance • Perception towards discrimination and unfairness
Hendriks et al., 2022[41]	Switzerland	Mixed methods	*N* = 69	Female (68%) Male (32%)	0–5 years: 18 (26%) 6–11 years: 24 (35%) 12–17 years: 27 (39%)	Time since end of treatment: < 5 years: 8 (12%) 6–15 years: 22 (33%) 16–25 years: 22 (33%) > 25 years: 14 (22%)	Survey: < 25 years: 28 (41%) 26–30 years: 12 (17%) 31–35 years: 13 (19%) > 35 years: 16 (23%)	No	Insurance Legal	• Limited eligibility for supplementary insurance • Late effects went unacknowledged
Holmqvist et al., 2010[42]	Sweden	Quantitative	*N* = 167	Female (52%) Male (48%)	6 (4.3/NR)	< 5 years: 77 (46%) 5–9 years: 59 (35%) 10–17 years: 31 (19%)	16–24 years: 60 (36%) 25–29 years: 43 (26%) 30–34 years: 38 (23%) ≥ 35 years: 26 (15%)	Yes (general population)	Financial	• Lower income • Sickness and/or disability compensation
Howard et al., 2014[43]	Canada	Quantitative and qualitative	*N* = 46	Female (56%) Male (44%)	8.5 (4.7/2.0–16.5)	NR	27 (7.4/16.0–46.0)	No	Financial Insurance Legal	• Not earning enough to cover living expenses • Parents continue to pay to prevent child from living in poverty • Difficulty to obtain disability allowance and loss of allowance • Restrictions to supplement disability allowance • Worries about what happens if or when they (parents) can no longer provide necessary financial support • Difficulties covering medical costs of their child • Struggling to pay for additional expenses like eyeglasses, hearing aids, dental work, vitamins, and prescription medications • Health coverage depending on certain conditions like living at home • Costs preventing parents from retiring • Difficulties obtaining different types of insurance • Employment discrimination (unfair barriers to, or denial of, assistance to accommodate special needs) • Theft, assault or fraud • Being denied public services: being denied public services, denied a divorce • Denied access to personal records or information • Unfairly accused of crimes by police, and threatened or coerced
Huang et al., 2019[9]	USA	Quantitative	*N* = 2811	Female (48%) Male (52%)	8.3 (5.6/1–24.8)	23.6 (8.1/10–48)	31.8 (8.4/18.3–64.5)	No	Financial Insurance	• Material hardship • Psychological hardship • Coping/behavioral hardship
Ingrand et al., 2022[44]	France	Mixed methods	*N* = 270	Female (49%) Male (51%)	NR	NR	NR (NR/18–32)	No	Legal	• Not talking about disease as grounds for dismissal • Discrimination at job
Johannesen et al., 2007[45]	Norway	Quantitative	*N* = 2944	NR	NR	NR	NR	No	Insurance Financial	• Health insurance benefits • Lower income
Kim et al., 2018[46]	South Korea	Qualitative	*N* = 15	Female (27%) Male (73%)	NR	Time since last treatment: 10.7 (NR/6–19)	20.7 (NR/15–28)	No	Legal	• Economic troubles • Social difficulties due to prejudice or discriminatory treatment • Feeling guilty because of familial economic difficulties • Social prejudice towards cancer patients • Discrimination when searching part-time job • Exemption from compulsory military service
Kirchhoff et al., 2010[47]	USA	Quantitative	*N* = 6339	Female (45%) Male (55%)	≤ 4 years: 1703 (27%) ≥ 4 years: 4636 (73%)	≤ 20 years: 1428 (22%) 21–30 years: 3979 (63%) > 30 932 (15%)	25–34 years: 3584 (56%) 35–44 years: 2196 (35%) ≥ 45 years: 559 (9%)	Yes (siblings)		• Health insurance coverage
Kirchhoff et al., 2013[48]	USA	Qualitative	*N* = 32	Female (50%) Male (50%)	NR	NR	NR (NR/25–46)	No	Financial Insurance Legal	• Inability to afford insurance without employer contribution • Not having insurance due to losing ESI upon becoming unemployed • Not having insurance due to not finding a job that offers insurance • Losing eligibility for parents’ ESI • Health insurance denial due to cancer history • Coverage only through spouses • Limitations in occupational choices because of insurance • Limited job mobility for survivors and spouses • Worries about losing ESI • Financial issues with affording health care • Financial barriers in their ability to pay for health care • Out-of-pocket costs • Inability to afford individual health insurance or secure employment that offered it • Insurance barriers
Kirchhoff et al., 2015[49]	USA	Quantitative	*N* = 698	Female (55%) Male (45%)	0–4 years: 365 (52%) 5–10 years: 143 (20%) 11–15 years: 109 (16%) 16–20 years: 81 (12%)	NR	22–29 years: 214 (30.7%) 30–39 years: 228 (32.7%) 40–62 years: 256 (36.7%)	Yes (siblings)	Insurance	• Receiving SSI benefits more common than in general population • Receiving DI benefits more common
Kirchhoff et al., 2018[50]	USA and Canada	Quantitative	*N* = 394	Female (46%) Male (54%)	0–5 years: 228 (47%) 6–10 years: 62 (20%) 11–15 years: 61 (19%) 16–20 years: 43 (14%)	NR	22–29 years: 114 (11%) 30–39 years: 137 (44%) 40–62 years: 143 (45%)	Yes (siblings)	Financial Insurance	• Job lock • Problems paying medical bills • Health insurance denial
Kirchhoff et al., 2024[51]	USA	Quantitative	*N* = 11535	Female (51%) Male (49%)	0–4 years: 4555 (40%) 5–9 years: 2534 (22%) 10–14 years: 2435 (21%) ≥ 15 years: 2011 (17%)	NR	18–25 years: 881 (7%) 26–29 years: 1565 (14%) 30–34 years: 2151 (19%) 35–39 years: 2539 (22%) 40–44 years: 2045 (18%) 45–49 years: 1375 (12%) ≥ 50 years: 979 (8%)	Yes (siblings)	Insurance	• Insurance coverage • Insurance instability • Underinsurance
Kuhlthau et al., 2016[52]	USA	Quantitative	*N* = 443	Female (69%) Male (31%)	NR	NR	38.3 (NR/NR)	Yes (general population)	Financial Insurance	• Problems with care accessibility and affordability (even when insured) • Problems with affording prescription medications, mental health services, dental care, eyeglasses, care from a specialist, and follow-up care in the previous 12 months • Delaying needed medical care • Problems affording care • Problems paying medical bills • Higher rates of worrying about medical bills • More often government-sponsored insurance
Leung et al., 2000[53]	USA	Quantitative	*N* = 77	Female (58%) Male (42%)	NR (NR/0.2–20.1)	Median length of follow-up since diagnosis: 16.7 (NR/10.1–23.5)	NR (NR/11.3–38.4)	No	Insurance	• Being uninsured
Löf et al., 2011[54]	Sweden	Quantitative	*N* = 51	Female (43%) Male (57%)	NR	Time since SCT: 19–24 years: 12 (4.9/5–22) 25–42 years: 20 (4.7/10–28)	19–24 years: 21 (1.6/19–24) 25–42 years: 30 (4.8/23–42)	Yes (general population)	Financial	• Income in poverty risk zone • Difficulty covering day-to-day expenses
Lönnerblad et al., 2023[55]	Sweden	Quantitative	*N* = 452	Female (48%) Male (52%)	0–5 years: 163 (36%) 6–9 years: 111 (25%) 10–14 years: 178 (39%)	NR	NR	Yes (general population)	Financial Insurance	• Receiving sickness or activity compensation
Maas et al., 2023[56]	The Netherlands	Quantitative	*N* = 1713	Female (49%) Male (51%)	6.8 (4.7/0.0–18)	29.2 (8.5/15.3–55.0)	36.0 (9.3/18.3–70.9)	Yes (non-participants)	Financial	• Financial problems
Miser et al., 2023[57]	Taiwan	Quantitative	*N* = 33105	Female (45%) Male (55%)	NR	Years of follow-up: Median 7.23 (NR/3.58–10.01)	Age at entry: Median 12 (NR/7–16)	Yes (healthy matched controls)	Financial	• Higher medical expenses • Higher annual outpatient expense
Mobley et al., 2022[5]	USA	Quantitative	*N* = 1106	Female (46%) Male (54%)	NR	NR	18–26 years: 635 (58%) 27–39 years: 471 (42%)	No	Insurance	• Insurance coverage change • Lost insurance coverage
Mody et al., 2008[58]	USA	Quantitative	*N* = 4151	Female (47%) Male (53%)	Median 4 (NR/0–21)	21.1 (NR/5–35)	Median 26	Yes (siblings)	Insurance	• Lower health insurance coverage
Mulrooney et al., 2008[59]	USA	Quantitative	*N* = 272	Female (54%) Male (46%)	7 (NR/0–20)	21.4 (NR/5–33)	28 (NR/10–49)	Yes (siblings)	Insurance	• Insurance coverage
Nagarajan et al., 2003[60]	USA	Quantitative	*N* = 694	Female (49%) Male (51%)	13.5 (NR/3–20)	15.8 (NR/6–28)	29.8 (NR/18–45)	Yes (siblings)	Insurance	• Health insurance coverage • Difficulty obtaining health insurance • Health insurance problems
Nathan et al., 2023[61]	USA	Quantitative	*N* = 3555	Female (52%) Male (48%)	Median 8.5 (NR/3.8–13.8)	NR	Median 40.5 (NR/35.0–47.5)	Yes (siblings)	Financial	• Material hardship • Psychological hardship • Coping/behavioral hardship • More likely to report being sent to debt collection • Problems paying medical bills • Foregoing needed medical care • Worry/stress about paying rent or mortgage or having enough money to buy nutritious meals
Nipp et al., 2017[13]	USA	Quantitative	*N* = 580	Female (53%) Male (47%)	NR	30.2 (4.6/NR)	22–29 years: 177 (11%) 30–39 years: 184 (41%) 40–62 years: 219 (48%)	Yes (siblings)	Financial	• Higher out-of-pocket medical costs • Problems paying medical bills • Deferring care, skipping a test, treatment, or follow-up • Worrying about affording health insurance • Taking a smaller dose of medication than prescribed • Thoughts of filing for bankruptcy
Nwachukwu et al., 2015[62]	USA	Quantitative	*N* = 121	Female (53%) Male (47%)	11.2 (NR/0.9–19.6)	11 (NR/2.5–41.4)	33.5 (NR/18–60)	Yes (reference sample of other study)	Financial	• Financial burden
Olson et al., 2011[8]	Canada	Quantitative	*N* = 111	Female (51%) Male (49%)	Median 7 (NR/3–11)	Median 25 (NR/19–28)	Median 31 (NR/26–36)	No	Legal	• Legal difficulties • Difficulty finding employment • Discrimination at work • Unfair termination of employment • Legal difficulties at school • Difficulty acquiring life, health or disability insurance
Otth et al., 2022[63]	Switzerland	Quantitative	*N* = 2154	Female (48%) Male (52%)	Median 10 (NR/4.5–14.3)	Median 16.1 (NR/10.6–21.9)	Median 24.6 (NR/20.1–31.4)	Yes (CNS tumors with other malignancies)	Financial	• Receiving disability pension
Ottaviani et al., 2013[64]	USA	Quantitative	*N* = 38	Female (63%) Male (37%)	13.2 (SE 0.7/3–19)	24.3 (SE 0.7/20–39)	37.9 (SE 1.1/22–52)	Yes (siblings)	Financial Insurance	• Financial dependence • Health insurance coverage
Park et al., 2005[65]	USA	Quantitative	*N* = 12358	Female (47%) Male (53%)	0–4 years: 5036 (41%) 5–9 years: 2730 (22%) 10–14 years: 2481 (20%) 15–20 years: 2111 (17%)	5–9 years: 1285 (10%) 10–14 years: 4658 (38%) 15–19 years: 3641 (29%) ≥ 20 years: 2774 (22%)	< 18 years: 3230 (26%) 18–21 years: 2114 (17%) 22–24 years: 1600 (13%) 25–34 years: 4225 (34%) ≥ 35 years: 1189 (10%)	Yes (siblings)	Insurance	• Health insurance coverage • Difficulties obtaining health insurance coverage • Being uninsured
Park et al., 2012[66]	USA	Qualitative	*N* = 39	Female (49%) Male (51%)	0–4 years: 24 (62%) 5–20 years: 15 (38%)	NR	≤ 30 years: 21 (54%) ≥ 30 years: 18 (46%)	No	Insurance Financial	• High out-of-pocket costs • Difficulty obtaining insurance • Minimizing need for care/avoiding care • Worries about future health care costs
Perez et al., 2018[4]	USA	Quantitative	*N* = 698	Female (55%) Male (45%)	0–5 years: 404 (46%) 6–10 years: 104 (19%) 11–15 years: 109 (20%) 16–20 years: 81 (15%)	Median 30.8	Median 39.4	Yes (siblings)	Financial Insurance	• Lower household income • Delaying mental health services due to cost • Mental health coverage • Gaps in insurance knowledge • Issues of underinsurance and health insurance literacy • Deferring needed care • Being uninsured • Being financially encumbered • Experiencing financial barriers to mental health care • Concerns affording mental health services • Economic barriers to care
Pickering et al., 2022[67]	Denmark	Quantitative	*N* = 2283	Female (48%) Male (52%)	9 (6/NR)	NR	20 or 30 (depending on group)	Yes (matched comparisons; malignant with benign tumors)	Financial	• Receiving disability pension • Lower income (also with social benefits) • Receiving social benefits • Higher health care costs • Higher psychiatric health care costs • Higher home care costs
Puhr et al., 2019[68]	Norway	Quantitative	*N* = 114	Female (58%) Male (42%)	9.4 (4.43/0.5–17)	Since treatment completion: 13.9 (5.61/2.6–25.1)	23.4 (3.5/NR)	Yes (healthy matched controls)	Financial Insurance	• More likely to previously and/or currently receive substantial government benefits
Pui et al., 2003[69]	USA	Quantitative	*N* = 584	Female (53%) Male (47%)	NR	Median 20 (NR/10–37)	Median 27 (NR/18–50)	Yes (general population)	Insurance	• Lack of health insurance • Denial of health insurance due to leukemia • Prohibitive premiums due to leukemia • Restrictions on health care plans due to leukemia • Difficulty obtaining health care • Not receiving needed care
Scholtes et al., 2019[70]	Germany	Quantitative	*N* = 270	Female (45%) Male (55%)	NR (NR/0.17–14.92)	NR (NR/11.92-34.08)	NR (NR/25.5–46.67)	Yes (general population)	Financial	• Financial difficulties
Warner et al., 2014[71]	USA	Qualitative	*N* = 17	Female (47%) Male (53%)	Median 15.5 (NR/2–20)	Median 32 (NR/11–36)	Median 45.5 (NR/27–56)	No	Financial Insurance	• Financial and insurance concerns • Financial difficulties with emergency care • Financial burden
Waters et al., 2024[72]	USA	Quantitative	*N* = 3503	Female (51%) Male (49%)	0–4 years: 1327 (41%) 5–9 years: 785 (23%) 10–14 years: 805 (21%) ≥ 15 years: 586 (15%)	NR	18–25 years: 236 (9%) 26–29 years: 316 (12%) 30–34 years: 543 (18%) 35–39 years: 721 (19%) 40–44 years: 615 (16%) 45–49 years: 503 (12%) ≥ 50 years: 569 (14%)	Yes (siblings)	Insurance	• Job lock
Yağci-Küpeli et al., 2013[73]	Turkey	Quantitative	*N* = 201	Female (37%) Male (63%)	Median 10 (NR/1–19)	Median 13.5 (NR/3–31)	Median 23 (NR/18–39)	Yes (general population)	Insurance	• Insurance status
Zebrack et al., 2010[74]	USA	Quantitative	*N* = 519	Female (52%) Male (48%)	11.3 (6.1/0–21)	15.4 (6.9/2–37)	26.7 (5.3/18–39)	No	Financial	• Financial problems

Where stratified age values were given, a value was calculated for the whole population

*ACA* Affordable Care Act, *CCS* childhood cancer survivors, *CNS* central nervous system, *DI* disability insurance, *ESI* employer-sponsored insurance, *IQR* interquartile range, *NHL* non-Hodgkin lymphoma, *NR* not reported, *RT* radiotherapy, *SADP* sickness absence and disability pension, *SCT* stem cell transplantation, *SE* standard error, *SSI* social security income

### Methodological quality of the publications

The overall quality of the included publications was 84%, ranging from 72 to 100%. The inter-rater reliability ranged from 0.73 to 1.00 for the different criteria, with a mean of 0.86 (standard deviation 0.08). With a weighted Kappa = 0.86, the interrater reliability showed almost perfect agreement [26] (Supplemental appendix C).

### Reported insurance, legal, and financial hardships

**Fig. 2:**
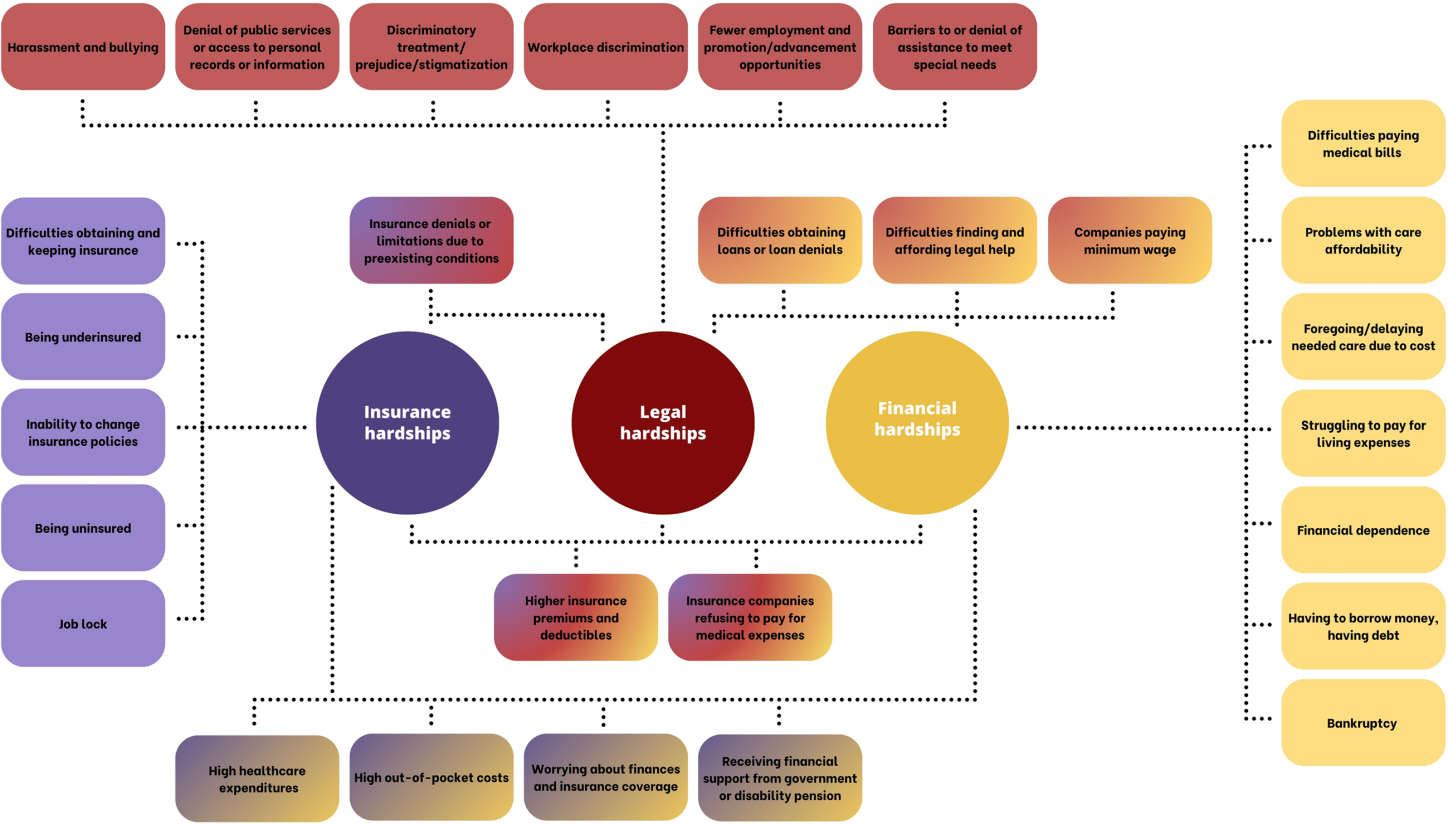
Insurance, legal, and financial hardships—an overview

CACS reported many different socio-bureaucratic hardships. In Fig. 2, we give an overview of insurance, legal, and financial hardships of CACS as covered in the included publications.

#### Health insurance coverage

Among publications included, several explicitly reported the percentage of CACS with health insurance. Percentages were reported mainly in publications from the US with *elective health insurance*. Reported health insurance coverage rates for adult CACS in the US ranged between 80 and 90% [5, 40, 53, 60, 64, 65, 69]. Nonetheless, compared to siblings, survivors were more likely to report underinsurance and less likely to perceive their coverage as stable [51]. In one US publication, almost half of insured survivors fulfilled the criteria of being underinsured [66]. Some publications found that CACS had significantly lower insurance rates compared to siblings [35, 37, 51, 58, 65], while others reported the opposite [59] or no difference [4, 64]. Differences in coverage varied across diagnoses [58], age groups [65], and ethnicity groups [29].

In several US publications [47, 48, 65, 69, 72] and one Canadian publication [43], some CACSs were able to receive (extended) health insurance through their own, spouses’, or parents’ employment [43, 47, 48, 65, 69, 72]. In one publication, health insurance was provided through an employer, spouse, or parent for almost 60% of participating CACS [69]. In another US publication, 56% of CACSs were covered through employment and 27% through a spouse or parent [65]. However, this coverage was often tied to certain conditions, such as receiving coverage only up to a certain age, requiring the survivor to live with their parents or be enrolled in post-secondary education, and depending on whether the parent [43] or spouse [48] continued to work. Being dependent on *employer-sponsored health insurance* led to “job lock” (i.e., self-reported inability of employee to leave a job freely due to limited portability of health insurance [50]) for some CACS and their spouses [48, 50, 72]. This phenomenon was reported only in US publications, occurring in almost a quarter of the CACS study participants and more frequently than in their siblings [50, 72]. CACSs reported being unable to afford insurance on their own, leading them to stay in jobs or limit their job search to positions offering insurance coverage [48]. This also affected spouses’ and parents’ occupational choices [43, 48, 72]. Other survivors were enrolled in federal or state-supported health plans (15.1% [69], 30.3% [52]), Medicaid (i.e., a government program in the US that helps cover medical costs for people with limited income and resources), or public assistance (12% [65]). To have government-sponsored insurance, Medicare (i.e., a federal health insurance program in the US for people age 65 and older and people with disabilities), Medicaid and public assistance was more likely among CACS compared to siblings [13, 51, 65] or comparisons [52].

From other countries, only a few publications have reported percentages of health insurance coverage of CACS. A Turkish publication has reported that the public social insurance rate, the most common method of obtaining health insurance coverage in Turkey, was 90.5% for CACSs [73]. In comparison to the general Turkish population, CACSs’ insurance rates were higher [73]. The lowest reported percentage for health insurance, with only 23.4% of CACS being insured, was reported in a publication from China [35]. CACSs were significantly less likely to be insured compared to their siblings [35].

Publications from European countries did not report percentages of health insurance coverage for CACS, presumably because of *compulsory health insurance* where everyone must be covered by some form of basic health insurance.

#### Difficulties obtaining and maintaining insurance

Even though most CACS reported being covered through compulsory or individual/employer-based *health insurance,* insurance-related difficulties were reported around the globe, also in European publications [3, 7, 14, 41]. In several countries, around 30% experienced difficulties in obtaining insurance [3, 8, 43, 60, 65, 66] and reported that they had been denied health insurance coverage in the past because of their illness history [66]. Some reported facing denials [6, 14, 48, 66, 69], exclusions, and restrictions [27, 41, 65, 69], such as ineligibility for *supplementary health insurance* [3, 41] or higher premiums and deductibles [6, 7, 65, 69]. Experiencing these kinds of difficulties was more likely for CACS than siblings [65].

Some publications reported on CACSs’ health insurance coverage changes, i.e., losing or gaining coverage [5, 29, 48]. Loss of health insurance was reported, for example, by US CACSs who became unemployed and therefore lost employer-sponsored insurance, or by younger CACSs who lost their parents’ insurance as they transitioned into adulthood [48]. CACSs who had supplementary health insurance before their cancer diagnosis were often no longer able to make changes to their insurance policy [3]. In one Swiss publication, almost half of the participating CACSs (45%) felt that their illness had negatively affected their lives with respect to insurance matters [41]. Nevertheless, the majority of respondents expressed satisfaction with the coverage provided by their basic health insurance [3, 66], except for administrative barriers [66], such as the inconvenience of obtaining reimbursement [3], and high costs (i.e., deductibles, copays, and premiums) [66].

Difficulties were also reported for other types of insurance [8, 43]. A French publication found that CACSs experienced challenges with *insurance for personal loans and home loans*, including rejections, exclusions, and higher premiums [14]. In this publication, 10.4% of CACS experienced difficulties obtaining a small loan and 30.1% of participants with obtaining a home loan [14]. Difficulties also occurred with *disability*, *risk*, *and life insurance*, as these options did not make financial sense, premiums were higher, and/or cancer was excluded from coverage [3, 7, 8]. A Dutch study reported problems obtaining funeral insurance [7]. Reasons for these difficulties were provided by different publications, including CACSs’ cancer history [7, 14, 41, 48, 69], preexisting conditions [6], or late effects [41]. CACSs also had lower proportions (21.3% vs. 50%) of life insurance coverage compared to their siblings [35].

#### Healthcare affordability and accessibility

CACSs faced large financial barriers in paying for health care [48]. Many CACSs reported having problems paying medical bills [13, 33, 52, 61], having limited means to cover substantial medical costs [43], and difficulties affording care [27, 48, 52]. Even when insured, CACS experienced problems with care affordability, including challenges in paying for prescription medications, dental care, eyeglasses, mental health services, and specialist and follow-up care [33, 52]. A majority of CACS paid for medical expenses out of pocket, causing hardship for both, them and their families [43, 48, 66]. CACS faced higher out-of-pocket costs than siblings [13] and reported higher health care, psychiatric care, and home care costs than matched comparisons [67]. Furthermore, CACS mentioned having to pay more or extra charges for their insurance premiums [6, 7, 65] or having enormous deductibles [6].

Several US publications reported that CACSs forgo necessary care [29, 52, 61, 66], do not adhere to, or delay recommended medical care [4, 11, 13, 38, 52] due to financial reasons, even when insured [38, 48, 52]. This included skipping tests, treatments, or follow-ups, or taking smaller doses of medication than prescribed [13, 52]. In one publication, more than one-third of CACS reported not seeing a doctor or going to the hospital when needed due to financial constraints [9]. In another publication, more than half of CACS reported experiencing at least one of the behavioral hardships mentioned above due to cost, which is more frequent than their peers [11]. One publication found no significant differences in mental health access, in terms of insurance coverage or cost-related delay of care, between CACSs and their siblings [4]. Some mental health coverage was reported by around two-thirds of both CACS and siblings [4].

#### Financial hardships and dependence on support

Financial burden experienced by CACS was reported in several of the included publications [9, 11, 27, 33, 39, 46, 61, 62, 70, 74]. CACS reported financial problems [56, 74] and economic troubles [46] and faced increased financial burden and difficulties compared to reference populations [33, 62, 70]. CACSs stated that their cancer experience had a significant negative impact on their financial situation, resulting in substantial material hardship [9] for up to 40% of surveyed CACS [11]. CACSs in a publication from the US were also more likely to report being sent to debt collection than their siblings [61].

In a Swedish publication, the income of 63% of participating CACS fell within the poverty risk zone, and 22% struggled to cover day-to-day expenses [54]. Half (50%) of a Canadian CACS cohort did not earn enough to cover most of their living expenses, as they only earned minimum wage—their parents supplemented their income to prevent them from living in poverty and to enable them to have a “reasonable” quality of life [43]. Similar issues were reported in other countries, with around 20% of CACS in a US publication reporting reduced spending on home improvement and basics such as food and clothing [33] and 55% of CACS in a Jordanian publication reporting a need for financial support to cover living expenses [27]. Insurance expenses have prevented some CACS from making progress in other areas of adult life, such as saving for a house deposit or for retirement [6].

The risk of financial dependency among CACS is increased [39]. CACSs were more likely to receive government benefits and disability pension than comparisons [28, 67, 68]. A Norwegian publication demonstrated that CACSs were four to five times more likely to receive financial support from the government than the cancer-free reference group [39]. Several publications from different countries reported that CACS were enrolled in supplemental security income (SSI) [49], disability insurance (DI) [3, 49], or receiving disability pension (DP) [28, 45, 63, 67], disability allowance [43], or government benefits due to sickness and/or disability [28, 32, 39, 42, 55, 67, 68]. In a Swedish publication, obtaining sickness or activity compensation was 11 times more likely for survivors than comparisons [55].

Even though such benefits can alleviate some financial burden for CACS, the maximum benefit payouts are rather low, sometimes even lower than the federal poverty level [49]. In a Canadian publication, 82% of participants indicated that the received disability allowance did not cover their living expenses [43]. In addition, these allowances come with restrictions, such as only being allowed to supplement them with very minimal earnings, making living independently unaffordable [43]. Moreover, CACS described experiencing difficulties when applying for disability pension [3] or allowance [43], with 27% of study participants rating the application process as difficult and 30% as very difficult [43]. The mentioned difficulties included understanding and completing the necessary paperwork, obtaining required assessments, a lack of understanding from officials, and convincing officials that CACS suffered from disability, resulting in stress, confusion, frustration, and the inability to initially obtain a disability allowance and the loss of allowance for some [3, 43].

#### Legal hardships and discriminatory challenges

Six of the included publications reported on legal hardships. In addition to difficulties with acquiring insurance (see the previous sections), some CACSs reported that insurance companies refused to pay their medical, dental, or disability-related expenses, forcing them to seek legal help [3, 43]. However, finding and affording legal assistance also proved to be difficult [3, 8].

Reported legal difficulties were common with 40.7% of CACS being affected in a Canadian publication [8]. Of the surveyed CACS, 22.2% reported legal difficulties at school [8]. However, the majority of legal issues were occupation-related, with 58.3% [8] and 45% [43] of Canadian CACS reporting such problems. These issues included fewer employment opportunities, difficulty finding employment, or unfair termination of employment [8, 46]. CACS from Canada, South Korea and France experienced workplace discrimination, such as unfair barriers to or denial of assistance for special needs [43, 44, 46], denial of equal mentoring opportunities [43], being paid minimum wage, and not being considered for promotion or advancement [43]. Discriminatory treatment after the disclosure of childhood cancer was also reported in relation to obtaining loans, even for French CACS with no present health conditions [14]. When trying to obtain small loans (10.4%) or a home loan (30.1%), CACSs faced difficulties such as rejection, exclusion, or higher premiums after disclosing their childhood cancer [14]. Instances of denial of public services or access to personal records or information were also reported [43].

#### Psychological aspects of insurance, legal, and financial hardships

Many of the included publications also reported on the psychological aspects of insurance, legal, and financial hardships [3, 9, 11, 13, 48, 52, 61, 66, 71]. Psychological financial hardship was reported by over half of the CACS in two North American publications [9, 11]. Other publications found that CACS experienced worry and stress about paying rent or mortgage [13, 61], affording (health) insurance [11, 13] and future health care costs [66], covering medical bills [52], and having enough money to buy nutritious meals [61] and household utilities [33], with some even having thoughts of filing for bankruptcy [13]. Economic difficulties due to childhood cancer also affected CACSs’ families, causing family problems [46]. Some CACSs reported feeling guilty because they believed the family issues, such as family problems or economic difficulties, were caused by them and their illness [46].

Other worries were insurance-related consequences of CACSs’ illness. In a Swiss publication, concerns were expressed regarding different types of insurance, spanning from basic health insurance to DI and private insurance [3]. CACS described feeling lost and stressed during the DI application process [3]. Others were worried about potentially negative attitudes toward being a DI-recipient [3]. Additional experiences included bullying and harassment in the workplace [43] and social difficulties due to prejudice or discriminatory treatment [46]. This reportedly made CACSs fearful and hesitant to disclose their situation to potential employers [44, 46] while at the same time worrying about the consequences of not disclosing [44]. Concerns about staying employed were mentioned by approximately half of the CACS in one US publication [11]; related incessant worries about losing ESI were reported by CACS in another US publication [48]. Loss of ESI was a concern because a reduction in working hours or job loss might lead to a potential loss of eligibility [48, 66]. CACS also feared insurance rescission, believing that insurers might cancel their policy retroactively because of their health status [48]. Another worry was that coverage of certain treatments or preventive care would be denied due to their cancer history [3, 48, 66].

### Hardship-related risk factors

Of the included publications, 25 identified potential risk factors for insurance, legal, and financial hardships. We provide an overview of the risk factors described (only statistically significant associations), ordered by cancer-related, treatment-related, demographic, socio-bureaucratic, health-related, and mental health-related factors in Table 2. Frequently mentioned risk factors were CNS tumors, being female, suffering from (chronic) health problems and having low household income, lack of insurance, and being unemployed.

**Table 2:** Overview of risk factors for insurance, legal, and financial hardships after childhood cancer as described in included publications

Reported risk factors	Hardship
Cancer-related factors	
Age at diagnosis	
- Younger age at diagnosis	Being uninsured [59, 65]
- Older age at diagnosis	Higher health care expenditures [30] Higher annual outpatient expenses [57]
Type of cancer	
- Brain and other CNS tumors/malignancies	Difficulties obtaining needed care [36] Higher health care and home care costs [67] Higher health care expenditures [30, 31] Higher annual outpatient expenses [57] Legal difficulties [8]
- Leukemia/hematologic malignancy	Financial hardship [11] Insurance denial [36] Being uninsured [59, 65]
- Non-Hodgkin’s lymphoma	Being uninsured [59, 65]
Subsequent cancers/tumor recurrence	Financial hardship [9, 62]
Treatment-related factors	
Chemotherapy	Higher medical costs [34] Financial hardship [61] Being uninsured [59, 65]
Irradiation/radiotherapy	Higher medical costs [34] Financial hardship [61] Being uninsured [65] Legal difficulties [8]
Surgery	Higher medical costs [34] Difficulties obtaining loans [14]
Demographic factors	
Age at study	
- Younger age at study	Financial hardship [61] Being uninsured [65]
- Older age at study	Higher health care expenditures [30, 31] Financial hardship [9, 11]
Gender	
- Female	Higher health care expenditures [30] Higher annual outpatient expenses [57] Higher out-of-pocket costs [13] Financial hardship [61] Job lock [50] Lower health insurance coverage (only females who were irradiated) [58]
- Male	Being uninsured [65]
Ethnicity	
- Being “black” (only reported in the US)	Higher out-of-pocket costs [13] Forgoing care [29]
Civil status	
- Being single	Financial hardship [61] Higher out-of-pocket costs [13]
- Being divorced/separated/widowed	Financial hardship [61] Being uninsured [65]
Socio-bureaucratic factors	
Household income	
- High	Higher medical costs [34]
- Low	Delaying and skipping needed care [4, 38] Difficulties affording care [38] Higher out-of-pocket costs [13] Financial hardship [9, 33, 74] Being uninsured [65]
Financial situation	
- Financial instability	Financial hardship [9]
- High percentage of income spent on out-of-pocket medical costs	Financial hardship [13] Thoughts of filing for bankruptcy [13] Delaying and skipping needed care/medication [13]
- Socioeconomic challenges	Financial hardship [27]
Educational attainment	
- Low	Financial hardship [9, 27, 33, 61, 70] Being uninsured [65]
Unemployment	Financial hardship [33, 74] Difficulties obtaining loans [14] Difficulties obtaining needed care [36] Higher out-of-pocket costs [13] Insurance denial [36] Being uninsured (social insurance) [73]
Socioeconomic status	
- High	Higher medical costs [34]
Insurance status	
- No insurance	Financial hardship [11, 33, 61] Higher out-of-pocket costs [13] Difficulties obtaining needed care [36] Postponing treatment due to cost [4] Forgoing care [29]
- Public insurance	Financial hardship [61]
- Private insurance	Higher out-of-pocket costs [13]
- Difficulties acquiring insurance	Financial hardship [9]
Legal difficulties	Financial hardship [8]
Health-related factors	
(Chronic) Health problems	Financial hardship [9, 27, 33, 74] Difficulties obtaining loans [14] Job lock [50] Higher out-of-pocket costs [13]
Hospitalization in the past year	Higher out-of-pocket costs [13]
Mental health-related factors	
Psychological/mental health problems	Financial hardship [9] Postponing treatment due to cost [4]

## Discussion

Our systematic review identified a considerable number of insurance, legal, and financial hardships reported by CACS and summarized the recently growing body of literature on these issues. The reported hardships show that these difficulties persist into survivorship. CACS reported long-term, intertwined hardships, such as higher insurance premiums and deductibles due to late effects, or insurance companies refusing to pay for medical expenses, resulting in problems with the affordability of needed care. The main problem areas described by CACS included difficulties with insurance companies, discrimination, and challenges affording necessary care. Survivors of CNS tumors, female CACS, and those reporting low household income, unemployment, or lack of insurance seem to be most at risk for these difficulties. Our review indicates that many of the reported hardships should be considered in the light of their interdependence.

Legal hardships were related to insurance, reimbursements, access to services, and employment, with workplace discrimination often reported. Since CACSs are more likely to be unemployed [75], experiencing unfair barriers or denial of assistance to meet special needs [43, 44, 46] may further complicate their entry into the labor market and increase the risk for unemployment. As unemployment has been reported as a risk factor for the hardships studied [13, 14, 36, 73, 74], especially for lack of insurance coverage and financial hardship, it is important to note the more difficult access. Compared to survivors of adult cancer, who have typically built careers before their cancer diagnosis and can rely on these careers when re-entering the workforce to regain some normalcy [76], CACSs may face difficulties from the start of their employment trajectory.

Insurance-related and financial hardships were strongly linked. In line with previous research and reviews [10, 77], our systematic review showed that CACSs are at an elevated risk for difficulties with affording necessary healthcare and paying medical bills. Costs, and ultimately medical problems, increase when treatments are not utilized due to lack of insurance coverage or high costs [78]. Our review demonstrated that, across different publications, obtaining new insurance or changing existing plans after diagnosis proves to be difficult for many CACSs. Despite many CACSs experiencing difficulties with insurance, most are insured. Publications reporting CACS being uninsured mainly stem from the US [58, 65], where health insurance is still mostly linked to employment [48]. However, the implementation of the Affordable Care Act (ACA) and the expansion of Medicaid have helped to reduce certain insurance coverage disparities in the US [51, 79, 80]. Protecting individuals with pre-existing conditions, who previously had limited affordable insurance options outside of employment or public insurance, is an important attainment of the ACA [51]. In other countries where health insurance is independently or governmentally organized and often compulsory, being uninsured does not pose a problem of that extent. In the Netherlands for instance, CACS cannot be refused insurance [56]. Though, it remains problematic that CACSs are often only eligible for general or basic insurance, not supplementary or dental insurance, unless they had comprehensive insurance coverage before their diagnosis [3, 41]. These problems not only affect the CACS population but also people living with other chronic health conditions [81]. As CACSs have an increased risk of chronic health conditions [82] and will need to utilize healthcare services more frequently in the long term [57], they may be even more vulnerable to these difficulties. Insurance coverage affects survivors’ ability to attend important follow-up visits [5] and receiving preventive services, treatment, and survival after a cancer diagnosis [83]. If we want CACSs to be able to take care of themselves and attend follow-up visits, care must be financially and bureaucratically manageable.

Furthermore, it is important to consider the possibility of survivors staying and/or becoming dependent on their parents or partners with regard to financial and insurance matters. The issue of job lock, which can affect both survivors and parents or spouses, represents a significant challenge in the context of insurance [48, 50, 72]. In future research, it could be beneficial to investigate whether this potentially also leads to survivors involuntarily remaining in cohabitating or marital arrangements. Another problematic observation of this systematic review is the impact of these socio-bureaucratic hardships on the psychological well-being of CACS [3, 9, 11, 13, 48, 52, 61, 71]. The included publications show that this is a substantial issue [9, 11] that should not be neglected.

### Implications for practice

This review revealed that insurance, legal, and financial hardships are often investigated as secondary outcomes and that their measurement is not standardized. While income, employment, insurance status, and socio-economic status are often surveyed in routinely applied questionnaires, long-term socio-bureaucratic hardships are surveyed to a lesser extent. In accordance with other researchers [10, 84], we believe that including these factors in serial assessments is important to better understand these hardships and their extent over time, identify risk groups, and determine when support is most needed.

Expanding or creating offers of support and guidance, or points of contact for socio-bureaucratic matters such as applying for disability or private insurance, filing insurance claims, or finding legal assistance throughout the cancer trajectory, can alleviate or potentially prevent hardship. Previous research shows that survivors have unmet needs in this regard [77, 85] or lack knowledge about laws [66], while our review shows that hardships often stem from bureaucratic hurdles, regulations, or complicated systems. Socio-bureaucratic support and information should be provided to CACS throughout their entire cancer trajectory and not just in the early stages of treatment. A variety of relevant materials for survivors are already available. For example, Hoffman [86] or Monaco and Smith [87] provide guidance for CACS on legal issues. When presented in a clear and accessible manner, such materials could serve as a basis for counseling and informing CACS on their rights, options, and scope for action.

For system-level interventions, such as reducing drug costs, altering reimbursement models, or designing value-based insurance [77, 88], applying collaborative efforts from research, policy, and practice would be crucial due to the complexity of the systems and the interdependence of hardships. An example of legislative initiatives adopted by several European countries is the recognition of a “Right to Be Forgotten” [14, 89, 90]. A legal stipulation that urges EU member states not to allow the use of health data related to oncological diseases when concluding insurance policies after a specified period [14, 89, 90]. Survivors no longer have to disclose their cancer history to insurers [14]. Implementing such initiatives is a step towards avoiding CACSs’ risk of discrimination.

### Study strengths and limitations

A major strength of this review is that it synthesizes evidence from the past > 20 years through a systematic and transparent approach, offering a broad and comprehensive perspective on insurance, legal, and financial hardships. By providing a holistic view of these three intertwined and mutually influencing topics, rather than examining them separately, we have gained a better understanding of the bigger picture of socio-bureaucratic hardships. However, there are some limitations to consider. One potential limitation is that we chose not to conduct additional searches of the referenced and grey literature, which may have resulted in the omission of some additional eligible publications. However, the number of publications included suggests that our search was extensive enough to provide a comprehensive review of the existing evidence. We recognize that grey literature in particular can be an important additional resource in systematic reviews, but the focus was on the available scientific evidence from original studies. Given the different health care, legal, and insurance systems, the included publications were embedded in; generalizability of the findings may be limited to each respective system. It is also noteworthy that some of the included publications were derived from the same long-term research projects.

## Conclusion

The many socio-bureaucratic hardships of CACS highlighted in this systematic review underscore the importance of not underestimating or neglecting long-term insurance, legal, and financial hardships. Our findings can serve as a basis for the enhancement and expansion of supportive care services addressing these difficulties by providing information and resources. With increasing numbers of survivors, it is crucial to offer tailored, long-lasting support to CACS with needs in these areas. To initiate effective interventions, collaborative efforts from research, policy, and practice are needed.

## Supplementary Information

Below is the link to the electronic supplementary material.

Supplementary file1 (PDF 861 KB)

## Data Availability

No datasets were generated or analysed during the current study.
