# Supplementary material for: Insurance, legal, and financial hardships of childhood and adolescent cancer survivors—a systematic review
Source: J Cancer Surviv. 2024 Nov 29;20(3):1055–80. doi: 10.1007/s11764-024-01710-3 (PMC13144184; doi:10.1007/s11764-024-01710-3)
Supplement: Supplementary file 1 — Supplementary file1 (PDF 861 KB) [file 11764_2024_1710_MOESM1_ESM.pdf]

Appendix to the manuscript [Journal of Cancer Survivorship]

Insurance, legal, and financial hardships of childhood and adolescent cancer survivors – a systematic review

Martina Ospelt[1], Pauline Holmer[1], Eva Maria Tinner[2,3], Luzius Mader[4], Manya Hendriks[1], Gisela Michel[1], Sonja Kälin[1*] & Katharina Roser[1*]

[1] University of Lucerne, Faculty of Health Sciences and Medicine, Lucerne, Switzerland

[2] Division of Pediatric Hematooncology, Inselspital, Bern University Hospital, University hospital Bern, Bern, Switzerland

[3] University Center of Internal Medicine, Kantonsspital Baselland, Liestal, Switzerland

[4] Cancer Registry Bern Solothurn, University of Bern, Bern, Switzerland

[*] shared last authorship

Corresponding author: Katharina Roser, Faculty of Health Sciences and Medicine, University of Lucerne, Alpenquai 4, 6005 Lucerne, Switzerland; katharina.roser@unilu.ch, +41 41 229 59 56

Overview:

Supplemental Appendix A – Search strategy

Supplemental Appendix B – Publication selection criteria

Supplemental Appendix C – Quality assessment of included publications

Supplemental appendix A – Search strategy

**PubMed**	
Search ID#	Search Terms
#1	"Insurance Coverage"[Mesh] OR "Insurance Benefits"[Mesh] OR "Financial Stress"[Mesh] OR "Cost of Illness"[Mesh:NoExp] OR "Health Expenditures"[Mesh:NoExp]
#2	economic[Title/Abstract] OR socioeconomic[Title/Abstract] OR financial[Title/Abstract] OR employment[Title/Abstract] OR unemployment[Title/Abstract] OR job[Title/Abstract] OR work ability[Title/Abstract] OR return to work [Title/Abstract] OR salar*[Title/Abstract] OR earning* [Title/Abstract] OR job lock [Title/Abstract] OR absenteeism[Title/Abstract] OR sick leave[Title/Abstract] OR sickness absence[Title/Abstract] OR early retirement[Title/Abstract] OR productivity loss*[Title/Abstract] OR treatment cost*[Title/Abstract] OR out-of-pocket[Title/Abstract] OR out of pocket[Title/Abstract] OR risk of poverty[Title/Abstract] OR risk-of-poverty[Title/Abstract] OR insurance[Title/Abstract] OR insur*[Title/Abstract] OR mortgage[Title/Abstract] OR loan*[Title/Abstract] OR reimburs*[Title/Abstract] OR discrimination[Title/Abstract] OR right to be forgotten[Title/Abstract] OR social welfare[Title/Abstract] OR legal[Title/Abstract] OR litigation[Title/Abstract] OR disability benefit*[Title/Abstract] OR social security benefit*[Title/Abstract] OR handicap allowance*[Title/Abstract] OR disability pension*[Title/Abstract]
#3	"Cancer Survivors"[Mesh] OR "Survival"[Mesh] OR "Parents"[Mesh] OR "Caregivers"[Mesh]
#4	surviv*[Title/Abstract] OR long-term surviv*[Title/Abstract] OR long term surviv*[Title/Abstract] OR parent*[Title/Abstract] OR mother*[Title/Abstract] OR father*[Title/Abstract] OR maternal[Title/Abstract] OR paternal[Title/Abstract] OR caregiver*[Title/Abstract] OR family care giver*[Title/Abstract] OR family caregiver*[Title/Abstract]
#5	"Child"[MesH] OR "Pediatrics"[Mesh] OR "Infant"[MesH] OR "Adolescent"[MesH]
#6	child*[Title/Abstract] OR infan*[Title/Abstract] OR adolescen*[Title/Abstract] OR newborn*[Title/Abstract] OR new born*[Title/Abstract] OR baby*[Title/Abstract] OR babies[Title/Abstract] OR neonat*[Title/Abstract] OR perinat*[Title/Abstract] OR postnat*[Title/Abstract] OR kids[Title/Abstract] OR kid[Title/Abstract] OR pediatric*[Title/Abstract] OR paediatric*[Title/Abstract] OR toddler*[Title/Abstract] OR teen*[Title/Abstract] OR boy[Title/Abstract] OR boys[Title/Abstract] OR girl*[Title/Abstract] OR juvenil*[Title/Abstract] OR youth*[Title/Abstract] OR young*[Title/Abstract] OR kindergar*[Title/Abstract] OR school*[Title/Abstract]
#7	childhood cancer[Title/Abstract] OR leukemia[Title/Abstract] OR leukemi*[Title/Abstract] OR leukaemi*[Title/Abstract] OR childhood ALL[Title/Abstract] OR AML[Title/Abstract] OR lymphoma[Title/Abstract] OR lymphom*[Title/Abstract] OR hodgkin[Title/Abstract] OR hodgkin*[Title/Abstract] OR T-cell[Title/Abstract] OR B-cell[Title/Abstract] OR non-hodgkin[Title/Abstract] OR sarcoma[Title/Abstract] OR sarcom*[Title/Abstract] OR Ewing*[Title/Abstract] OR osteosarcoma[Title/Abstract] OR osteosarcom*[Title/Abstract] OR wilms tumor[Title/Abstract] OR wilms*[Title/Abstract] OR nephroblastom*[Title/Abstract] OR neuroblastoma[Title/Abstract] OR neuroblastom*[Title/Abstract] OR rhabdomyosarcoma[Title/Abstract] OR rhabdomyosarcom*[Title/Abstract] OR teratoma[Title/Abstract] OR teratom*[Title/Abstract] OR hepatoma[Title/Abstract] OR hepatom*[Title/Abstract] OR hepatoblastoma[Title/Abstract] OR hepatoblastom*[Title/Abstract] OR PNET[Title/Abstract] OR PNET*[Title/Abstract] OR medulloblastoma[Title/Abstract] OR medulloblastom*[Title/Abstract] OR neuroectodermal tumors, primitive[Title/Abstract] OR retinoblastoma[Title/Abstract] OR retinoblastom*[Title/Abstract] OR meningioma[Title/Abstract] OR meningiom*[Title/Abstract] OR glioma[Title/Abstract] OR gliom*[Title/Abstract] OR pediatriconcology[Title/Abstract] OR paediatric oncology[Title/Abstract] OR childhood tumor[Title/Abstract] OR childhood tumour[Title/Abstract] OR childhood tumors[Title/Abstract] OR childhood tumours[Title/Abstract] OR brain tumor*[Title/Abstract] OR brain tumour*[Title/Abstract] OR brain neoplasms[Title/Abstract] OR central nervous system neoplasm[Title/Abstract] OR central nervous system neoplasms[Title/Abstract] OR central nervous system tumor*[Title/Abstract] OR central nervous system tumour*[Title/Abstract] OR brain cancer*[Title/Abstract] OR brain neoplasm*[Title/Abstract] OR intracranial neoplasm*[Title/Abstract]
#8	all [sb] "Animals"[Mesh] NOT "Humans"[Mesh]

#9	("2000/01/01"[Date - Publication] : "2023"[Date - Publication])
#10	#1 OR #2
#11	#3 OR #4
#12	#5 OR #6
#13	#10 AND #11 AND #12 AND #7
#14	#13 NOT #8
#15	#14 AND #9

Note: Combined search for survivors and parents. The hardships experienced by parents are discussed in another publication.

**CINAHL**	
Search ID#	Search Terms
S1	(MH "Insurance+") OR (MM "Financial Stress")
S2	TI(economic OR socioeconomic OR financial OR employment OR unemployment OR job OR (work N3 ability) OR ("return to work") OR salar* OR earning* OR (job W1 lock) OR absenteeism OR (sick W1 leave) OR (sickness W1 absence) OR (early W1 retirement) OR (productivity N3 loss*) OR (treatment N3 cost*) OR out-of-pocket OR ("out of pocket") OR ("risk of poverty") OR risk-of-poverty OR insurance OR insur* OR mortgage OR loan* OR reimburs* OR discrimination OR ("right to be forgotten") OR (social W1 welfare) OR legal OR litigation OR (disability W1 benefit*) OR ("social security benefit*") OR (handicap W1 allowance*) OR (disability W1 pension*))
S3	AB(economic OR socioeconomic OR financial OR employment OR unemployment OR job OR (work N3 ability) OR ("return to work") OR salar* OR earning* OR (job W1 lock) OR absenteeism OR (sick W1 leave) OR (sickness W1 absence) OR (early W1 retirement) OR (productivity N3 loss*) OR (treatment N3 cost*) OR out-of-pocket OR ("out of pocket") OR ("risk of poverty") OR risk-of-poverty OR insurance OR insur* OR mortgage OR loan* OR reimburs* OR discrimination OR ("right to be forgotten") OR (social W1 welfare) OR legal OR litigation OR (disability W1 benefit*) OR ("social security benefit*") OR (handicap W1 allowance*) OR (disability W1 pension*))
S4	(MM "Cancer Survivors") OR (MM "Survival") OR (MH "Parents+") OR (MM "Caregivers")
S5	TI(surviv* OR ("long-term surviv*") OR ("long term surviv*") OR parent* OR mother* OR father* OR maternal OR paternal OR caregiver* OR ("family care giver*") OR (family W1 caregiver*))
S6	AB(surviv* OR ("long-term surviv*") OR ("long term surviv*") OR parent* OR mother* OR father* OR maternal OR paternal OR caregiver* OR ("family care giver*") OR (family W1 caregiver*))
S7	(MH “Child+”) OR (MH “Infant+”) OR (MH “Adolescence+”) OR (MH “Pediatrics+”)
S8	TI(child* OR infan* OR adolescen* OR newborn* OR (new W1 born*) OR baby* OR babies OR neonat* OR perinat* OR postnat* OR kids OR kid OR pediatric* OR paediatric* OR toddler* OR teen* OR boy OR boys OR girl* OR juvenil* OR youth* OR young* OR kindergar* OR school*)
S9	AB(child* OR infan* OR adolescen* OR newborn* OR (new W1 born*) OR baby* OR babies OR neonat* OR perinat* OR postnat* OR kids OR kid OR pediatric* OR paediatric* OR toddler* OR teen* OR boy OR boys OR girl* OR juvenil* OR youth* OR young* OR kindergar* OR school*)
S10	(MM “Childhood Neoplasms”)
S11	TI((childhood W1 cancer) OR leukemia OR leukemi* OR leukaemi* OR (childhood W1 ALL) OR AML OR lymphoma OR lymphom* OR hodgkin OR hodgkin* OR T-cell OR B-cell OR non-hodgkin OR sarcoma OR sarcom* OR Ewing* OR osteosarcoma OR osteosarcom* OR (wilms W1 tumor) OR wilms* OR nephroblastom* OR neuroblastoma OR neuroblastom* OR rhabdomyosarcoma OR rhabdomyosarcom* OR teratoma OR teratom* OR hepatoma OR hepatom* OR hepatoblastoma OR hepatoblastom* OR PNET OR PNET* OR medulloblastoma OR medulloblastom* OR ("neuroectodermal tumors, primitive") OR retinoblastoma OR retinoblastom* OR meningioma OR meningiom* OR glioma OR gliom* OR pediatriconcology OR (paediatric W1 oncology) OR (childhood W1 tumor) OR (childhood W1 tumour) OR (childhood W1 tumors) OR (childhood W1 tumours) OR (brain W1 tumor*) OR (brain W1 tumour*) OR (brain W1 neoplasms) OR ("central nervous system neoplasm") OR ("central nervous system neoplasms") OR ("central nervous system tumor*")

	OR ("central nervous system tumour*") OR (brain W1 cancer*) OR (brain W1 neoplasm*) OR (intracranial W1 neoplasm*))
S12	AB((childhood W1 cancer) OR leukemia OR leukemi* OR leukaemi* OR (childhood W1 ALL) OR AML OR lymphoma OR lymphom* OR hodgkin OR hodgkin* OR T-cell OR B-cell OR non-hodgkin OR sarcoma OR sarcom* OR Ewing* OR osteosarcoma OR osteosarcom* OR (wilms W1 tumor) OR wilms* OR nephroblastom* OR neuroblastoma OR neuroblastom* OR rhabdomyosarcoma OR rhabdomyosarcom* OR teratoma OR teratom* OR hepatoma OR hepatom* OR hepatoblastoma OR hepatoblastom* OR PNET OR PNET* OR medulloblastoma OR medulloblastom* OR ("neuroectodermal tumors, primitive") OR retinoblastoma OR retinoblastom* OR meningioma OR meningiom* OR glioma OR gliom* OR pediatriconcology OR (paediatric W1 oncology) OR (childhood W1 tumor) OR (childhood W1 tumour) OR (childhood W1 tumors) OR (childhood W1 tumours) OR (brain W1 tumor*) OR (brain W1 tumour*) OR (brain W1 neoplasms) OR ("central nervous system neoplasm") OR ("central nervous system neoplasms") OR ("central nervous system tumor*") OR ("central nervous system tumour*") OR (brain W1 cancer*) OR (brain W1 neoplasm*) OR (intracranial W1 neoplasm*))
S13	(MH "Animals+" not MH "Humans+")
S14	PY 2000-2023 PY 2023-2024 (for search update)
S15	S1 OR S2 OR S3
S16	S4 OR S5 OR S6
S17	S7 OR S8 OR S9
S18	S10 OR S11 OR S12
S19	S15 AND S16 AND S17 AND S18
S20	S19 NOT S13
S21	S20 AND S14 AND Limitations (Peer Reviewed) applied<br>AND Limitations Human<br>AND Limitations 2000-2023 2023-2024 (for search update)

Note: Combined search for survivors and parents. The hardships experienced by parents are discussed in another publication.

**PsychInfo**	
Search ID#	Search Terms
S1	MAINSUBJECT.EXACT.EXPLODE("Insurance") OR MAINSUBJECT.EXACT.EXPLODE("Financial Strain")
S2	economic OR socioeconomic OR financial OR employment OR unemployment OR job OR "work ability" OR "return to work" OR salar* OR earning* OR "job lock" OR absenteeism OR "sick leave" OR "sickness absence" OR "early retirement" OR "productivity loss*" OR "treatment cost*" OR out-of-pocket OR "out of pocket" OR "risk of poverty" OR risk-of-poverty OR insurance OR insur* OR mortgage OR loan* OR reimburs* OR discrimination OR "right to be forgotten" OR "social welfare" OR legal OR litigation OR "disability benefit*" OR "social security benefit*" OR "handicap allowance*" OR "disability pension*"
S3	MAINSUBJECT.EXACT.EXPLODE("Survivors") OR MAINSUBJECT.EXACT.EXPLODE("Parents") OR MAINSUBJECT.EXACT.EXPLODE("Caregivers")
S4	surviv* OR "long-term surviv*" OR "long term surviv*" OR parent* OR mother* OR father* OR maternal OR paternal OR caregiver* OR "family care giver*" OR "family caregiver*"
S5	MAINSUBJECT.EXACT.EXPLODE("Pediatrics") OR MAINSUBJECT.EXACT.EXPLODE("Adolescent Health")
S6	child* OR infan* OR adolescen* OR newborn* OR new born* OR baby* OR babies OR neonat* OR perinat* OR postnat* OR kids OR kid OR pediatric* OR paediatric* OR toddler* OR teen* OR boy OR boys OR girl* OR juvenil* OR youth* OR young* OR kindergar* OR school*
S7	"childhood cancer" OR leukemia OR leukemi* OR leukaemi* OR "childhood ALL" OR AML OR lymphoma OR lymphom* OR hodgkin OR hodgkin* OR T-cell OR B-cell OR non-hodgkin OR sarcoma OR sarcom* OR Ewing* OR osteosarcoma OR osteosarcom* OR "wilms tumor" OR wilms* OR nephroblastom* OR neuroblastoma OR neuroblastom* OR

	rhabdomyosarcoma OR rhabdomyosarcom* OR teratoma OR teratom* OR hepatoma OR hepatom* OR hepatoblastoma OR hepatoblastom* OR PNET OR PNET* OR medulloblastoma OR medulloblastom* OR "neuroectodermal tumors, primitive" OR retinoblastoma OR retinoblastom* OR meningioma OR meningiom* OR glioma OR gliom* OR pediatriconcology OR "paediatric oncology" OR "childhood tumor" OR "childhood tumour" OR "childhood tumors" OR "childhood tumours" OR "brain tumor*" OR "brain tumour*" OR "brain neoplasms" OR "central nervous system neoplasm" OR "central nervous system neoplasms" OR "central nervous system tumor*" OR "central nervous system tumour*" OR "brain cancer*" OR "brain neoplasm*" OR "intracranial neoplasm*"
S8	[S1] OR [S2]
S9	[S3] OR [S4]
S10	[S5] OR [S6]
S11	[S8] AND [S9] AND [S10] AND [S7]
S12	[S11] AND decade: 2000 - 2009; 2010 - 2019; 2020 - 2029
S13	[S12] AND Limitations (only scientific journals) applied
S14	[S13] AND Limitations (verified by experts) applied

Note: Combined search for survivors and parents. The hardships experienced by parents are discussed in another publication.

**Scopus**	
Search ID#	Search Terms
S1	TITLE-ABS-KEY(economic OR socioeconomic OR financial OR employment OR unemployment OR job OR {work ability} OR {return to work} OR salar* OR earning* OR job lock OR absenteeism OR {sick leave} OR {sickness absence} OR {early retirement} OR {productivity loss*} OR {treatment cost*} OR out-of-pocket OR {out of pocket} OR {risk of poverty} OR risk-of-poverty OR insurance OR insur* OR mortgage OR loan* OR reimburs* OR discrimination OR {right to be forgotten} OR {social welfare} OR legal OR litigation OR {disability benefit*} OR {social security benefit*} OR {handicap allowance*} OR {disability pension*})
S2	TITLE-ABS-KEY (surviv* OR {long-term surviv*} OR {long term surviv*} OR parent* OR mother* OR father* OR maternal OR paternal OR caregiver* OR {family care giver*} OR {family caregiver*})
S3	TITLE-ABS-KEY (child* OR infan* OR adolescen* OR newborn* OR {new born*} OR baby* OR babies OR neonat* OR perinat* OR postnat* OR kids OR kid OR pediatric* OR paediatric* OR toddler* OR teen* OR boy OR boys OR girl* OR juvenil* OR youth* OR young* OR kindergar* OR school*)
S4	TITLE-ABS-KEY ({childhood cancer} OR leukemia OR leukemi* OR leukaemi* OR {childhood ALL} OR all OR aml OR lymphoma OR lymphom* OR hodgkin OR hodgkin* OR t-cell OR b-cell OR non-hodgkin OR sarcoma OR sarcom* OR Ewing* OR osteosarcoma OR osteosarcom* OR {wilms tumor} OR wilms* OR nephroblastom* OR neuroblastoma OR neuroblastom* OR rhabdomyosarcoma OR rhabdomyosarcom* OR teratoma OR teratom* OR hepatoma OR hepatom* OR hepatoblastoma OR hepatoblastom* OR pnet OR PNET* OR medulloblastoma OR medulloblastom* OR {neuroectodermal tumors, primitive} OR retinoblastoma OR retinoblastom* OR meningioma OR meningiom* OR glioma OR gliom* OR pediatriconcology OR {paediatric oncology} OR {childhood tumor} OR {childhood tumour} OR {childhood tumors} OR {childhood tumours} OR {brain tumor*} OR {brain tumour*} OR {brain neoplasms} OR {central nervous system neoplasm} OR {central nervous system neoplasms} OR {central nervous system tumor*} OR {central nervous system tumour*} OR {brain cancer*} OR {brain neoplasm*} OR {intracranial neoplasm*})
S5	PUBYEAR > 1999 AND PUBYEAR < 2024 AND (LIMIT-TO (EXACTKEYWORD , "Human") OR LIMIT-TO (EXACTKEYWORD , "Humans"))
S6	S1 AND S2 AND S3 AND S4
S7	S6 AND S5

Note: Combined search for survivors and parents. The hardships experienced by parents are discussed in another publication.

Supplemental appendix B – Publication selection criteria

	Inclusion criteria	Exclusion criteria
Language	• All languages	
Publication type	• Published since 2000<br>• Published in a peer-reviewed scientific journal	
Study type	• Human studies<br>• Quantitative study or qualitative study (empirical studies)	• Review article, editorial, conference abstract, and commentary
Population	• Adult survivors of childhood or adolescent cancer (age ≥16)<br>• Diagnosis of childhood or adolescent cancer (≥75 % of sample or separate analyses)<br>• Age at diagnosis <18 (≥75 % of sample, or separate analyses)<br>• Time since diagnosis >2 years (≥75 % of sample, or separate analyses)	• Survivors of cancer other than childhood or adolescent cancer<br>• <75 % of sample with childhood or adolescent cancer (e.g., other childhood diseases)
Outcomes	• Survivors' hardships:<br>▪ Insurance<br>▪ Legal<br>▪ Financial	• Studies not using survivors' financial, legal, and insurance-related hardships as outcomes (e.g. simply described in Demographics Table 1).

Supplemental appendix C – Quality assessment of included publications

		1) Theoretical or conceptual underpinning to the research		2) Statement of research aim/s		3) Clear description of research setting and target population		4) The study design is appropriate to address the stated research aim/s		5) Appropriate sampling to address the research aim/s		6) Rationale for choice of data collection tool/s	
**Authors short**	**Year**	**Rater 1**	**Rater 2**	**Rater 1**	**Rater 2**	**Rater 1**	**Rater 2**	**Rater 1**	**Rater 2**	**Rater 1**	**Rater 2**	**Rater 1**	**Rater 2**
Al-Rawashdeh et al.	2024	3	3	3	3	3	3	3	3	2	2	3	3
Baecklund et al.	2022	2	3	3	3	3	3	3	3	3	3	2	2
Baedke et al.	2021	3	3	3	3	3	3	3	3	3	2	3	2
Bejarano-Quisoboni et al.	2022	2	2	3	2	3	3	3	3	3	2	3	2
Bejarano-Quisoboni et al.	2023	3	3	3	3	3	3	3	3	2	2	2	2
Boman et al.	2010	3	2	3	3	3	3	3	3	3	3	3	3
Buchbinder et al.	2023	3	3	3	3	3	3	3	3	3	2	3	3
Carlson-Green	2009	2	3	3	2	3	2	3	3	2	1	2	3
Chae et al.	2020	3	2	3	3	3	3	3	3	3	3	3	2
Chan et al.	2020	2	3	3	3	3	3	3	3	2	2	3	2
Clemens et al.	2017	2	2	3	3	3	3	3	3	3	1	3	2
Crom et al.	2007	2	2	3	3	3	3	3	3	2	2	2	2
Dumas et al.	2017	3	2	3	3	3	2	3	3	3	2	3	2
Fair et al.	2021	3	2	3	3	3	3	3	3	3	2	3	3
Fauer et al.	2024	3	2	2	3	3	3	3	3	2	2	3	3
Fiala	2021	3	2	1	2	3	3	3	3	3	3	3	2
Gunnes et al.	2016	3	2	3	3	3	3	3	3	3	3	3	2
Guy et al.	2016	2	2	2	3	3	3	3	3	3	3	3	2
Hendriks et al.	2021	2	3	3	3	3	3	3	3	3	2	2	2
Hendriks et al.	2022	2	3	3	3	3	3	3	3	2	2	2	3

Holmqvist et al.	2010	2	2	2	2	3	3	3	3	3	3	3	2
Howard et al.	2014	3	3	3	3	3	3	3	3	2	3	3	3
Huang et al.	2019	3	3	3	3	3	3	3	3	3	2	3	2
Ingrand et al.	2022	2	3	3	3	3	3	3	3	2	3	1	2
Johannesen et al.	2007	3	2	3	3	3	3	3	3	3	3	3	2
Kim et al.	2018	3	1	3	3	2	3	3	3	3	3	2	2
Kirchhoff et al.	2024	3	2	3	3	2	2	3	3	2	2	2	2
Kirchhoff et al.	2018	2	2	3	3	3	2	3	3	3	2	3	2
Kirchhoff et al.	2010	2	3	3	3	3	3	3	3	2	3	1	3
Kirchhoff et al.	2013	2	3	3	3	3	3	3	3	3	3	2	3
Kirchhoff et al.	2015	2	3	3	2	2	3	3	3	2	2	2	2
Kuhlthau et al.	2016	3	2	3	3	3	3	3	3	3	3	3	2
Leung et al.	2000	2	2	3	3	3	3	3	3	2	2	2	2
Löf et al.	2011	3	2	3	3	3	3	3	3	3	2	3	2
Lönnerblad et al.	2023	3	2	3	3	3	2	3	3	2	2	2	2
Maas et al.	2023	3	3	3	3	3	3	3	3	3	3	3	3
Miser et al.	2023	2	3	3	2	3	3	3	3	2	3	0	3
Mobley et al.	2022	3	3	3	3	3	3	3	3	2	2	2	2
Mody et al.	2008	2	2	3	3	3	3	3	3	2	2	2	2
Mulrooney et al.	2008	2	2	3	3	2	3	3	3	2	2	1	2
Nagarajan et al.	2003	2	3	2	3	3	3	3	3	2	2	2	2
Nathan et al.	2022	2	3	2	2	3	3	3	3	2	2	3	3
Nipp et al.	2017	2	2	3	3	3	3	3	3	3	3	1	3
Nwachukwu et al.	2015	2	2	3	3	3	3	3	3	2	3	3	3
Olson et al.	2011	2	2	3	1	3	3	3	3	2	2	3	3
Otth et al.	2022	2	2	3	3	3	3	3	3	2	3	2	3
Ottoviani et al.	2013	1	1	2	3	3	3	3	3	2	2	1	2
Park et al.	2005	2	3	3	3	3	3	3	3	2	2	2	2
Park et al.	2012	3	3	3	3	3	3	3	3	3	3	3	3
Perez et al.	2018	2	3	3	3	3	3	3	3	2	1	3	2
Pickering et al.	2022	2	3	2	3	2	3	3	3	2	3	2	3

Puhr et al.	2021	3	3	3	3	3	3	3	3	2	3	1	3
Pui et al.	2003	2	2	2	2	3	3	3	3	2	2	2	2
Scholtes et al.	2019	3	3	3	3	3	3	3	3	2	2	1	2
Warner et al.	2014	2	2	3	3	3	3	3	3	3	3	3	3
Waters et al.	2024	2	1	2	3	3	3	3	3	3	3	2	3
Yağci-Küpeli et al.	2013	2	3	3	3	3	3	3	3	2	2	2	2
Zebrack et al.	2010	3	3	2	3	3	3	3	3	2	3	3	3

		7) The format and content of data collection tool is appropriate to address the stated research aim/s		8) Description of data collection procedure		9) Recruitment data provided		10) Justification for analytic method selected		11) The method of analysis was appropriate to answer the research aim/s		12) Evidence that the research stakeholders have been considered in research design or conduct	
**Authors short**	**Year**	**Rater 1**	**Rater 2**	**Rater 1**	**Rater 2**	**Rater 1**	**Rater 2**	**Rater 1**	**Rater 2**	**Rater 1**	**Rater 1**	**Rater 2**	**Rater 1**
Al-Rawashdeh et al.	2024	3	3	3	3	3	3	3	3	3	3	1	2
Baecklund et al.	2022	3	3	3	3	2	3	3	2	3	3	0	0
Baedke et al.	2021	3	3	2	3	3	3	3	3	3	3	0	0
Bejarano-Quisoboni et al.	2022	3	3	2	3	1	2	2	3	3	3	0	0
Bejarano-Quisoboni et al.	2023	3	3	3	2	2	3	2	2	3	3	0	0
Boman et al.	2010	3	3	2	3	2	3	3	3	3	3	0	0
Buchbinder et al.	2023	3	3	2	2	2	3	3	3	3	3	0	0
Carlson-Green	2009	3	3	3	2	2	1	3	3	3	3	0	0
Chae et al.	2020	3	3	2	3	3	3	2	3	3	3	0	0
Chan et al.	2020	3	3	3	2	3	1	3	3	3	3	2	1
Clemens et al.	2017	3	3	3	2	2	1	3	1	3	3	0	0
Crom et al.	2007	3	3	3	2	2	3	3	3	3	3	0	0
Dumas et al.	2017	3	3	1	2	3	2	3	3	3	3	0	0

Fair et al.	2021	3	3	3	3	3	2	3	3	3	3	3	1
Fauer et al.	2024	3	3	3	3	2	3	2	3	3	3	0	2
Fiala	2021	3	3	2	3	2	1	3	3	3	3	0	0
Gunnes et al.	2016	3	3	3	3	3	3	3	3	3	3	0	0
Guy et al.	2016	3	3	2	3	3	1	3	3	2	3	0	0
Hendriks et al.	2021	3	3	3	3	3	3	3	3	3	3	0	0
Hendriks et al.	2022	3	3	3	3	3	3	3	3	3	3	0	2
Holmqvist et al.	2010	3	3	2	3	2	3	3	3	3	3	0	0
Howard et al.	2014	3	3	3	3	3	3	2	3	3	3	2	0
Huang et al.	2019	3	3	3	3	3	3	3	3	3	3	0	0
Ingrand et al.	2022	3	3	3	3	3	3	0	3	3	3	1	2
Johannesen et al.	2007	3	3	3	3	3	3	2	1	3	2	0	0
Kim et al.	2018	3	3	3	3	3	2	3	2	3	3	2	2
Kirchhoff et al.	2024	3	3	3	3	2	2	3	3	3	3	0	0
Kirchhoff et al.	2018	3	3	3	3	3	3	3	3	3	3	0	0
Kirchhoff et al.	2010	2	3	1	3	3	3	3	3	3	3	0	0
Kirchhoff et al.	2013	3	3	3	3	3	3	2	1	3	3	2	2
Kirchhoff et al.	2015	3	3	3	2	2	2	3	3	3	3	0	0
Kuhlthau et al.	2016	3	3	3	3	3	2	3	3	3	3	0	0
Leung et al.	2000	3	3	3	2	3	3	3	1	3	3	0	0
Löf et al.	2011	3	3	3	3	3	3	3	3	3	3	0	0
Lönnerblad et al.	2023	3	3	3	3	2	2	2	3	3	3	0	0
Maas et al.	2023	3	3	3	3	3	3	3	3	3	3	0	0
Miser et al.	2023	3	3	2	3	3	3	0	3	2	3	0	0
Mobley et al.	2022	3	3	3	3	2	3	3	3	3	3	0	0
Mody et al.	2008	3	3	3	2	2	3	3	2	3	3	0	0
Mulrooney et al.	2008	3	3	3	3	3	3	3	3	3	3	0	0
Nagarajan et al.	2003	3	3	3	2	3	3	3	2	3	2	0	0
Nathan et al.	2022	3	3	3	3	3	3	3	3	3	3	0	0
Nipp et al.	2017	3	3	3	3	3	3	1	3	3	3	1	0
Nwachukwu et al.	2015	3	3	2	3	3	3	1	3	3	3	0	0

Olson et al.	2011	3	3	3	2	3	2	3	2	3	3	2	2
Otth et al.	2022	3	3	0	3	1	3	2	3	3	3	0	0
Ottoviani et al.	2013	2	3	3	3	3	3	1	3	3	3	0	0
Park et al.	2005	3	3	3	3	3	3	3	3	3	3	0	0
Park et al.	2012	3	3	3	3	3	3	3	3	3	3	3	3
Perez et al.	2018	3	3	3	2	3	3	3	3	3	3	2	2
Pickering et al.	2022	3	3	1	3	2	3	2	3	3	3	0	0
Puhr et al.	2021	3	3	3	3	3	3	3	3	3	3	0	0
Pui et al.	2003	3	3	3	3	3	2	3	3	3	3	0	0
Scholtes et al.	2019	3	3	2	2	3	3	3	3	3	3	0	0
Warner et al.	2014	3	3	3	3	3	3	0	0	3	3	0	0
Waters et al.	2024	3	3	3	3	2	3	3	3	3	3	0	0
Yağci-Küpeli et al.	2013	3	3	3	2	3	3	3	1	3	3	0	0
Zebrack et al.	2010	3	3	2	2	3	3	3	3	3	3	0	0

		**13) Strengths and limitations critically discussed**		**Total**		**Quality (% rounded)**		**QuADS Mean Raters**
**Authors short**	**Year**	**Rater 1**	**Rater 2**	**Total Rater 1**	**Total Rater 2**	**Quality Rater 1 (%)**	**Quality Rater 2 (%)**	
Al-Rawashdeh et al.	2024	3	3	36	37	92%	95%	94%
Baecklund et al.	2022	3	3	33	34	85%	87%	86%
Baedke et al.	2021	3	3	35	34	90%	87%	89%
Bejarano-Quisoboni et al.	2022	3	3	31	31	79%	79%	79%
Bejarano-Quisoboni et al.	2023	3	3	32	32	82%	82%	82%
Boman et al.	2010	3	3	34	35	87%	90%	89%
Buchbinder et al.	2023	3	3	34	34	87%	87%	87%
Carlson-Green	2009	3	0	32	26	82%	67%	75%
Chae et al.	2020	3	3	34	34	87%	87%	87%

Chan et al.	2020	3	3	36	32	92%	82%	87%
Clemens et al.	2017	2	2	33	26	85%	67%	76%
Crom et al.	2007	3	3	32	32	82%	82%	82%
Dumas et al.	2017	3	3	34	30	87%	77%	82%
Fair et al.	2021	3	2	39	33	100%	85%	93%
Fauer et al.	2024	3	3	32	36	82%	92%	87%
Fiala	2021	3	3	32	31	82%	79%	81%
Gunnes et al.	2016	3	3	36	34	92%	87%	90%
Guy et al.	2016	3	3	32	32	82%	82%	82%
Hendriks et al.	2021	3	3	34	34	87%	87%	87%
Hendriks et al.	2022	3	3	33	37	85%	95%	90%
Holmqvist et al.	2010	3	3	32	33	82%	85%	84%
Howard et al.	2014	2	3	35	36	90%	92%	91%
Huang et al.	2019	3	3	36	34	92%	87%	90%
Ingrand et al.	2022	0	1	27	35	69%	90%	80%
Johannesen et al.	2007	1	2	33	30	85%	77%	81%
Kim et al.	2018	3	1	36	31	92%	79%	86%
Kirchhoff et al.	2024	3	3	32	31	82%	79%	81%
Kirchhoff et al.	2018	1	2	33	31	85%	79%	82%
Kirchhoff et al.	2010	3	3	29	36	74%	92%	83%
Kirchhoff et al.	2013	3	2	35	35	90%	90%	90%
Kirchhoff et al.	2015	3	3	31	31	79%	79%	79%
Kuhlthau et al.	2016	3	3	36	33	92%	85%	89%
Leung et al.	2000	3	3	33	30	85%	77%	81%
Löf et al.	2011	3	2	36	32	92%	82%	87%
Lönnerblad et al.	2023	3	3	32	31	82%	79%	81%
Maas et al.	2023	3	3	36	36	92%	92%	92%
Miser et al.	2023	3	3	26	35	67%	90%	79%
Mobley et al.	2022	3	3	33	34	85%	87%	86%
Mody et al.	2008	3	3	32	31	82%	79%	81%
Mulrooney et al.	2008	2	3	30	33	77%	85%	81%

Nagarajan et al.	2003	3	3	32	31	82%	79%	81%
Nathan et al.	2022	3	3	33	34	85%	87%	86%
Nipp et al.	2017	3	3	32	35	82%	90%	86%
Nwachukwu et al.	2015	3	3	31	35	79%	90%	85%
Olson et al.	2011	2	2	35	30	90%	77%	84%
Otth et al.	2022	3	3	27	35	69%	90%	80%
Ottoviani et al.	2013	1	2	25	31	64%	79%	72%
Park et al.	2005	1	2	31	33	79%	85%	82%
Park et al.	2012	3	3	39	39	100%	100%	100%
Perez et al.	2018	3	3	36	34	92%	87%	90%
Pickering et al.	2022	2	3	26	36	67%	92%	80%
Puhr et al.	2021	3	3	33	36	85%	92%	89%
Pui et al.	2003	0	0	29	28	74%	72%	73%
Scholtes et al.	2019	3	3	32	33	82%	85%	84%
Warner et al.	2014	3	3	32	32	82%	82%	82%
Waters et al.	2024	1	1	30	32	77%	82%	80%
Yağci-Küpeli et al.	2013	1	2	31	30	79%	77%	78%
Zebrack et al.	2010	3	3	33	35	85%	90%	88%

Note: **Weighted Cohen's Kappa=.86** (Criterion 1 = .73; Criterion 2 = .88; Criterion 3 = .86; Criterion 4 = 1.0; Criterion 5 = .81; Criterion 6 = .80; Criterion 7 = .97; Criterion 8 = .83; Criterion 9 = .77; Criterion 10 = .82; Criterion 11 = .93; Criterion 12 = .93; Criterion 13 = .90)
